# Chromosome Translocations, Gene Fusions, and Their Molecular Consequences in Pleomorphic Salivary Gland Adenomas †

**DOI:** 10.3390/biomedicines10081970

**Published:** 2022-08-14

**Authors:** Göran Stenman, Andre Fehr, Alena Skálová, Vincent Vander Poorten, Henrik Hellquist, Lauge Hjorth Mikkelsen, Nabil F. Saba, Orlando Guntinas-Lichius, Carlos Miguel Chiesa-Estomba, Mattias K. Andersson, Alfio Ferlito

**Affiliations:** 1 Sahlgrenska Center for Cancer Research, Department of Pathology, University of Gothenburg, SE-405 30 Gothenburg, Sweden; 2 Department of Pathology, Faculty of Medicine, Charles University, 301 66 Plzen, Czech Republic; 3 Bioptic Laboratory, Ltd., 305 99 Plzen, Czech Republic; 4 Otorhinolaryngology-Head and Neck Surgery, University Hospitals Leuven, 3000 Leuven, Belgium; 5 Department of Oncology, Section Head and Neck Oncology, KU Leuven, 3000 Leuven, Belgium; 6 Faculty of Medicine and Biomedical Sciences (FMCB), University of Algarve, 8005-139 Faro, Portugal; 7 Algarve Biomedical Center Research Institute (ABC-RI), 8005-139 Faro, Portugal; 8 Northern Lincolnshire and Goole NHS Foundation Trust, Lincoln 999039, UK; 9 Department of Pathology, Copenhagen University Hospital, 2100 Copenhagen, Denmark; 10 Department of Hematology and Medical Oncology, The Winship Cancer Institute, Emory University, Atlanta, GA 30322, USA; 11 Department of Otorhinolaryngology, Jena University Hospital, 07747 Jena, Germany; 12 Department of Otorhinolaryngology-Head and Neck Surgery, Hospital Universitario Donostia, Biodonostia Research Institute, University of Deusto, 20014 San Sebastian, Spain; 13 Coordinator of the International Head and Neck Scientific Group, 35122 Padua, Italy

**Keywords:** pleomorphic adenoma, chromosome translocation, chromosome 8q12, chromosome 12q13-15, gene fusion, PLAG1, HMGA2, IGF2, diagnostic biomarker, therapeutic target

## Abstract

Salivary gland tumors are a heterogeneous group of tumors originating from the major and minor salivary glands. The pleomorphic adenoma (PA), which is the most common subtype, is a benign lesion showing a remarkable morphologic diversity and that, upon recurrence or malignant transformation, can cause significant clinical problems. Cytogenetic studies of >500 PAs have revealed a complex and recurrent pattern of chromosome rearrangements. In this review, we discuss the specificity and frequency of these rearrangements and their molecular/clinical consequences. The genomic hallmark of PA is translocations with breakpoints in 8q12 and 12q13-15 resulting in gene fusions involving the transcription factor genes *PLAG1* and *HMGA2*. Until recently, the association between these two oncogenic drivers was obscure. Studies of the Silver–Russel syndrome, a growth retardation condition infrequently caused by mutations in *IGF2*/*HMGA2*/*PLAG1*, have provided new clues to the understanding of the molecular pathogenesis of PA. These studies have demonstrated that HMGA2 is an upstream regulator of *PLAG1* and that HMGA2 regulates the expression of IGF2 via *PLAG1*. This provides a novel explanation for the 8q12/12q13-15 aberrations in PA and identifies IGF2 as a major oncogenic driver and therapeutic target in PA. These studies have important diagnostic and therapeutic implications for patients with PA.

## 1. Introduction

Salivary gland tumors are a large and heterogeneous group of neoplasms originating from the major salivary glands (the parotid, submandibular, and sublingual glands) as well as from the numerous minor salivary glands in the oral mucosa and upper aerodigestive tract. The diversity of histologic subtypes originating from these glands is remarkable and there are more than 30 known histological subtypes of benign and malignant salivary gland tumors of which the pleomorphic adenoma (PA) is the most common [1,2]. Mucoepidermoid carcinomas and adenoid cystic carcinomas are the two most common malignant salivary gland tumors.

PA is a benign rather slow-growing epithelial tumor of which approximately three-quarters are located in the parotid gland. They can occur in all age groups but most often in the 5th and 6th decades and with a slight female predominance [3,4]. The etiology is unknown but there is a reported association to radiation exposure [5,6]. PAs are usually encapsulated and are recognized for their morphological diversity [7]. They are composed of ductal epithelial and myoepithelial cells growing in a variety of patterns in a stroma that is often mucoid, myxoid, hyalinized, or chondroid. Some tumors may be predominantly cellular with only scanty stroma. Mitotic figures are rare. PA frequently shows metaplastic changes, of which squamous metaplasia is the most common. Oncocytic and sebaceous metaplasia are other frequent metaplastic changes that can confuse in the diagnostic work up [8]. Because of the broad morphological spectrum of PAs, they may sometimes mimic malignancy and show morphological/architectural overlap with for example adenoid cystic carcinoma and polymorphous adenocarcinoma [8].

PAs may undergo malignant transformation to carcinoma-ex-PA (CXPA). The risk of malignant transformation is greater in long-standing or recurrent tumors and occurs in about 12% of recurrent PAs [9]. CXPA is usually a high-grade tumor with rapid and aggressive growth and frequent recurrences and metastases. The carcinoma component can be of any type, most often salivary duct carcinoma (SDC), myoepithelial carcinoma (MECA), or adenocarcinoma NOS.

PA was the first benign epithelial tumor in which characteristic chromosome translocations and gene fusions were identified [10,11,12,13,14,15]. Recurrent t(3;8)(p21;q12) and t(9;12)(p23-24;q14-15) translocations were shown to be early cytogenetic events [16]. Subsequent molecular cloning of the translocation breakpoints in these and other translocations in PA revealed that they consistently result in gene fusions. The prime molecular targets of these translocations are the transcription factor genes *PLAG1* (Pleomorphic Adenoma Gene 1; located in 8q12) and *HMGA2* (High Mobility Group AT-Hook 2; located in 12q14-15) [11,12,13,17,18,19]. Notably, the early cytogenetic and molecular genetic studies of translocations in PA during the 80s and 90s paved the way for the discovery of a diagnostically relevant gene fusion network in PA as well as in several other subtypes of salivary gland tumors [18,20,21]. The aim of this paper is to review the comprehensive literature on chromosome translocations/rearrangements and gene fusions in PA and to discuss their molecular consequences and clinical significance.

## 2. The Cytogenetic Landscape of PA

### 2.1. Overview of the Chromosomal Pattern in PA

**Figure 1:**
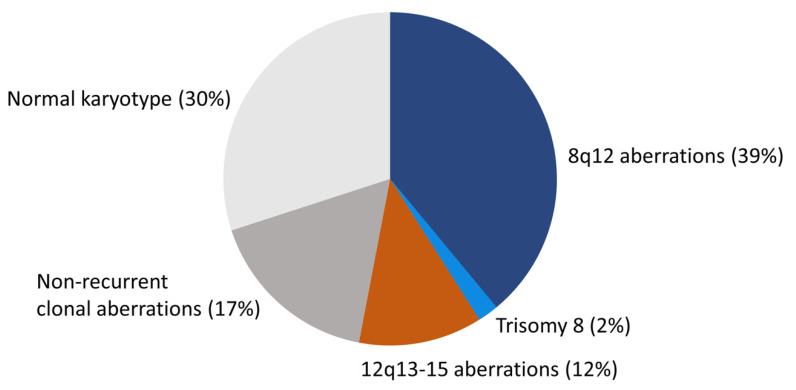
The cytogenetic landscape of PA. Pie chart showing the different cytogenetic subgroups and their frequencies in PA.

PA is cytogenetically the best-studied benign epithelial neoplasm. Studies by in particular two groups have revealed a consistent pattern of chromosome translocations/rearrangements in about 70% of the cases [10,14,16,19,22,23,24,25,26,27,28,29,30,31,32,33,34,35,36]. The remaining 30% have shown normal karyotypes without visible chromosome rearrangements (Figure 1). Detailed analysis of high-resolution banded chromosomes have failed to detect cytogenetic alterations in these PAs. However, molecular analyses have unequivocally demonstrated that they have submicroscopic changes, including inversions, small insertions, or deletions resulting in gene fusions (see below) [13,19,37,38].

The *Mitelman Database of Chromosome Aberrations and Gene Fusions in Cancer* contains cytogenetic data from 354 salivary gland PAs with abnormal karyotype [36]. Since tumors with normal karyotypes (≈30%) are not included in the database, the total number of PAs that have been cytogenetically characterized is more than 500 [10,14,16,19,22,23,24,25,26,27,28,29,30,31,32,33,34,35]. PAs with an abnormal karyotype form four major subgroups: (i) tumors with translocations/rearrangements involving chromosome 8q12 (≈39%), (ii) tumors with translocations/rearrangements involving chromosome 12q13-15 (≈12%), (iii) tumors with complete or partial trisomy 8 (≈2%), and (iv) tumors with non-recurrent clonal aberrations without involvement of 8q12 or 12q13-15 (≈17%) (Figure 1).

The t(3;8)(p21;q12) translocation is the most common aberration in the 8q12 subgroup (≈47%), followed by t(8;9)(q12;p22-24) or the related ins(9;8)(p23;q11q12) (≈10%), t(5;8)(p13-15;q12) (≈4%), t(6;8)(p21-22;q12) (≈3%), t(8;10)(q12;q22-23) (≈2%), and t(8;15)(q12;q26) (≈2%). There is also a variety of 8q12 variant translocations with other less frequent translocation partners.

The t(9;12)(p21-23;q13-15) and the related ins(9;12)(p23:q14q15) or ins(12;9)(q15;p24p22) (≈16%) are the most common aberrations in the 12q13-15 subgroup, followed by t(3;12)(p12-14;q14-15) (≈5%), t(6;12)(q21-23;q14-15) (≈5%), inv(12)(p11-13q13-14) (≈5%), and inv(12)(q13-15q23-24) (≈5%). Similar to the 8q12 subgroup, there are also several different 12q13-15 variant translocations with other less frequent translocation partners.

Trisomy 8 forms the smallest subgroup of PAs. In 65% of these cases, +8 is seen as the sole aberration. In the remaining cases, it is seen together with other aberrations, in particular 12q13-15 rearrangements. In addition to trisomy 8, there are also several cases reported with partial gain of one to four copies of chromosome 8 in the form of ring chromosomes r(8)(p12q12) (see below) [39]. Complete or partial trisomy 8 is the only major numerical aberration found in PA.

The fourth major subgroup of PAs consists of tumors with non-recurrent clonal aberrations without the involvement of 8q12 or 12q13-15. This is a heterogeneous group of tumors some of which have a single translocation as the sole deviation whereas others have more complex karyotypes with both structural and numerical aberrations. The molecular pathogenesis of these tumors remains to be elucidated. However, previous studies have indicated that at least some of these cases have cryptic rearrangements involving 8q12 [38].

### 2.2. Double Minute Chromosomes and Homogeneously Staining Regions

A subset of PAs with 12q13-15 aberrations, in particular those with del(12)(q13q15), show cytogenetic evidence of gene amplification in the form of double minute chromosomes (dmin) and homogeneously staining regions (hsr) [35,40,41,42]. Detailed molecular characterization of these aberrations have shown that the prime targets of the amplifications are the *HMGA2* and *MDM2* genes [42]. Other less frequently co-amplified genes are *WIF1*, *TSPAN31*, *CDK4*, and *GLI1*. Notably, in several cases a cryptic *HMGA2::WIF1* fusion gene was highly amplified and overexpressed [42]. Previous studies have also suggested that PAs with amplification of *MDM2* and possibly also other driver genes in 12q have an increased risk of malignant transformation [42,43,44]. However, this hypothesis needs to be confirmed by studies of additional cases of CXPA and by in vitro transformation experiments.

### 2.3. Ring Chromosomes and Dicentric Chromosomes

**Figure 2:**
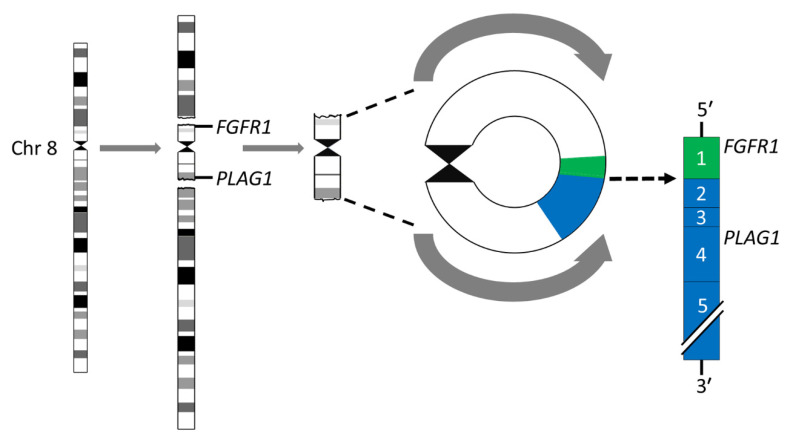
Ring chromosome 8 in PA. Schematic illustration of the formation of the ring chromosome r(8)(p12q12.1) and the resulting *FGR1::PLAG1* gene fusion in which exon 1 of *FGR1* (green) is fused to exon 2 of *PLAG1* (blue).

Breakage-fusion-bridge (BFB) cycles [45,46] is a mechanism that generates chromosome variability and mitotically unstable chromosome aberrations such as ring chromosomes and dicentric chromosomes [47]. Ring chromosomes have been found in ≈8% of karyotypically aberrant PAs [24,26,35,36,39,48]. The most common rings are derived from chromosomes 8 and 5, in that order of frequency. The rings may vary in both size and number in a given tumor. Notably, we have previously shown that the r(8)(p12q12.1) consists of a pericentromeric segment with recurrent breakpoints in *FGFR1* in 8p12 and *PLAG1* in 8q12.1 with amplification and overexpression of an *FGFR1::PLAG1* gene fusion (Figure 2) [39]. Importantly, this fusion has also been shown to be enriched 15-fold in myoepithelial carcinoma-ex-PA (MECAXPA) compared to PA, suggesting that amplification and overexpression of the *FGFR1::PLAG1* fusion may be a potential biomarker for malignant transformation of PA [49].

Rings derived from other chromosomes have so far not resulted in gene fusions, but instead losses of segments of for example 8p, 5p, 5q, and/or 6q [39], indicating the presence of putative tumor suppressor genes in these regions. Dicentric chromosomes are rarer than rings and are seen in only 1.5% of cytogenetically abnormal PAs. The majority of these involve chromosome 8 with breakpoints in 8q12.

### 2.4. Polyclonal Aberrations in Radiation-Associated PAs

Cytogenetic studies on radiation-associated PAs are rare. To the best of our knowledge, there are only two such cases reported in which PA developed in patients previously treated with radiotherapy for tuberculous lymphadenitis in the neck [5,50]. Both cases were cytogenetically polyclonal with a variety of mainly unique structural aberrations, but without involvement of the PA-specific breakpoints 8q12 and 12q13-15. Subsequent molecular analyses revealed that both tumors had activation of *PLAG1* and *HMGA2* and one of the cases had a cryptic *CTNNB1::PLAG1* fusion [6]. Studies of these two unique cases demonstrate that polyclonal, radiation-associated PAs develop as a result of very similar basic molecular mechanisms as sporadic PAs with monoclonal karyotypes.

## 3. The Gene Fusion Landscape of PA

### 3.1. The Transcription Factor Gene PLAG1 Is the Target of Translocations and Rearrangements of 8q12 in PA

**Figure 3:**
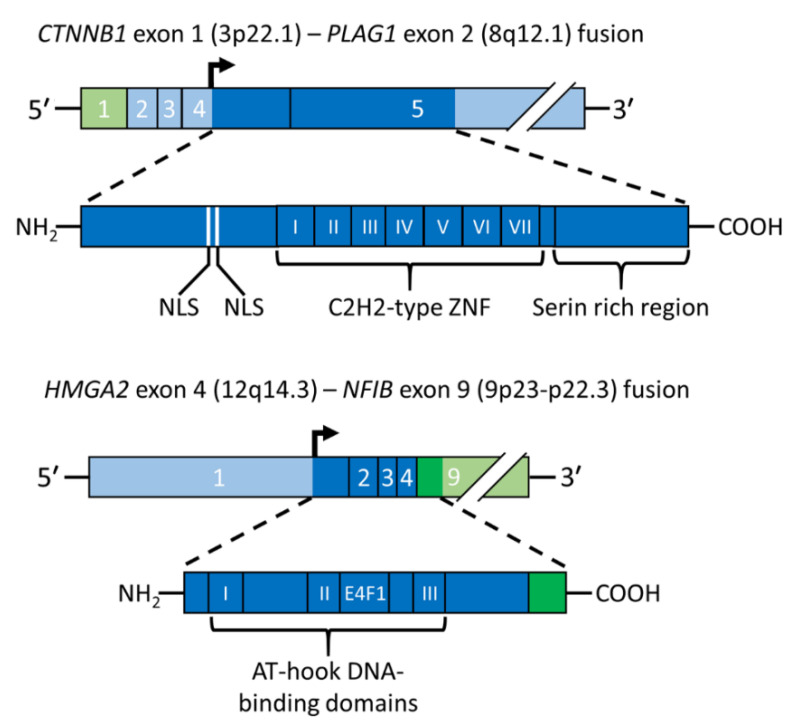
Schematic illustration of gene fusions in PA. The *CTNNB1::PLAG1* fusion gene and the encoded fusion protein is depicted in the upper panel and the *HMGA2::NFIB* fusion gene and the encoded fusion protein in the lower panel. Coding exons are shown in darker green and blue and the translation start site is indicated by an arrow. AT-hook, DNA binding motif; E4F1, E4F1-domain; NLS, nuclear localization signal; ZNF, zinc finger.

PA was the first benign epithelial tumor in which a tumor type-specific translocation was shown to result in a gene fusion. Positional cloning of the breakpoints in the t(3;8)(p21;q12) translocation revealed that it generates a fusion between the *PLAG1* gene in 8q12 and *CTNNB1* gene in 3p21 [11] (Figure 3, upper panel). The *PLAG1* oncogene encodes a developmentally regulated transcription factor that is highly expressed in various fetal tissues, but whose expression is low or absent in adult tissues, including normal salivary gland [11,37]. PLAG1 has seven canonical C2H2 zinc finger domains in the N-terminal part of the protein and a serine-rich transcriptional activation domain in its C-terminal part [11,51]. The PLAG1 oncoprotein is involved in the regulation of crucial cellular processes such as transcriptional regulation, growth control, cell proliferation, and apoptosis [52,53]. Its fundamental role in growth control is further emphasized by studies of knockout mice showing that the pre- and postnatal growth of *Plag1^−/−^* mice is significantly retarded [54]. The finding that the growth factor IGF2 is a major downstream target of PLAG1 [55] also agrees with this finding. PLAG1 binds the IGF2 P3 promoter and aberrantly activates its expression in PAs overexpressing PLAG1 [55]. Other known PLAG1 targets are *BCL2*, *CDKN1C*, *CRABP2*, *CYTL1*, *EFNB1*, *SMARCD3*, and *TSPAN4* [53].

*CTNNB1* is the most common fusion partner gene to *PLAG1* in PA. *CTNNB1* encodes β-catenin, a ubiquitously expressed protein involved in the regulation of cell–cell adhesion, cell proliferation, and differentiation. β-catenin acts as an intracellular signal transducer in the Wnt signaling pathway and is inhibited by the tumor suppressor protein APC [56]. *CTNNB1* is mutated in a variety of solid tumors [56,57].

The t(3;8) translocation in PA results in promoter swapping between *PLAG1* and *CTNNB1* leading to activation of *PLAG1* expression [11] (Figure 3, upper panel). The *CTNNB1::PLAG1* fusion was the first example of promoter swapping in solid tumors. The breakpoints in *PLAG1* and *CTNNB1* occur in the non-coding regions of both genes, leading to exchange of regulatory control elements with the coding sequences preserved. Subsequent studies of other translocations have confirmed that ectopic overexpression of a normal PLAG1 oncoprotein due to promoter swapping is a recurrent theme in PA. Thus, also the t(8;9)/ins(9;8) and t(5;8), which are the second and third most common aberrations in the 8q12 subgroup of PAs, result in exchange of regulatory sequences between *PLAG1* and the *NFIB* and *LIFR* genes, respectively [17,37]. Notably, *NFIB* is also a fusion partner of *HMGA2* in PA and of *MYB* in salivary gland adenoid cystic carcinomas (see below) [13,37,58,59].

**Figure 4:**
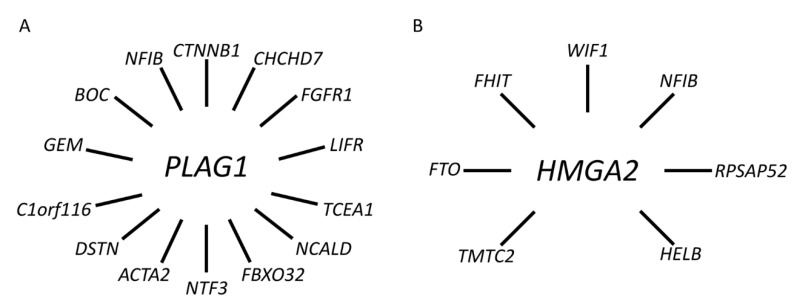
Gene fusion network in PA. (**A**) *PLAG1* and its 14 known fusion partner genes. (**B**) *HMGA2* and its seven known fusion partner genes.

To date, we and others have identified a network of *PLAG1* fusions in PA involving at least 11 additional fusion partner genes, including *CHCHD7*, *FGFR1*, *TCEA1*, *NCALD*, *FBXO32*, *NTF3*, *ACTA2*, *DSTN*, *C1orf116*, *GEM*, and *BOC* [38,39,60,61,62] (Figure 4A and Supplementary Table S1).

It should be emphasized that several of these fusions, including e.g., *CHCHD7::PLAG1* and *TCEA1::PLAG1*, are cryptic fusions generated by intrachromosomal rearrangements [38]. For example, *CHCHD7* and *PLAG1* are located head-to-head only 500 bp apart and due to a paracentric inversion *PLAG1* is activated by promoter swapping. The common denominator for all fusion partners is that they are either ubiquitously expressed or highly expressed in normal salivary gland. Importantly, the fusions are generated both by cytogenetically visible translocations/aberrations and cryptic intra- and interchromosomal rearrangements [38]. The latter are often, but not exclusively, found in PAs with an apparently normal karyotype [19]. The diversity of chromosome 8q12 aberrations in PA suggest that additional *PLAG1* fusion partners are likely to be found.

In summary, the 8q12 translocations/aberrations in PA result in ectopic activation of *PLAG1* expression due to promoter swapping with a variety of different, mostly ubiquitously expressed, fusion partner genes. The oncogenic activity of PLAG1 is partly due to activation of IGF2-signaling, suggesting that the IGF2-pathway is a potential therapeutic target in PA.

### 3.2. HMGA2 Is the Target Gene of Translocations and Rearrangements of 12q13-15 in PA

Parallel to the discovery of the *PLAG1* gene as the target of the 8q12 aberrations in PA [11] we could demonstrate by positional cloning that the target of the 12q13-15 rearrangements in PA was the *HMGA2* gene (previously known as *HMGIC*) [12,13]. *HMGA2* is a member of the non-histone high-mobility group (HMG) protein gene family and encodes an architectural transcription factor [63,64,65,66]. HMG-proteins are essential components of enhanceosomes and are involved in the regulation of gene transcription, recombination, and chromatin structure. The HMGA2 protein has three AT-hook DNA-binding domains in its N-terminus, a spacer domain, and an acidic C-terminus. Examples of known down-stream targets of *HMGA2* are *PLAG1* and the cell cycle regulator *CCNA1* [67,68].

Detailed genomic analyses of the 3p and 12q breakpoints in a PA with a complex karyotype, including an ins(3;12)(p14.2;q14q15), revealed an *HMGA2*-fusion linking the first three exons (encoding the DNA-binding domains) of *HMGA2* to the two last coding exons of the *FHIT* gene in 3p14.2 [12]. The deduced HMGA2::FHIT fusion protein consists of the N-terminal part of HMGA2, including the three DNA-binding domains, fused to the C-terminal part of FHIT which encodes the last 31 amino acids of the protein. Subsequent molecular analysis of a PA with an ins(9;12) confirmed that also this rearrangement involves *HMGA2* and results in a fusion of the 3′-part of the gene linked to the last coding exon of the *NFIB* gene which only encodes five amino acids [13]. Notably, it was also shown that a PA with an apparently normal karyotype had a cryptic ins(9;12) generating an *HMGA2::NFIB* fusion. Later studies confirmed that also the t(9;12)(p21-23;q13-15), which together with the ins(9;12) variant are the most common aberrations in the 12q13-15 subgroup of PAs, result in *HMGA2::NFIB* gene fusions [37] (Figure 3, lower panel). Notably, *NFIB* is so far the only fusion partner gene that is shared between *PLAG1* and *HMGA2*. *NFIB* is also a fusion partner to the *MYB* oncogene in adenoid cystic carcinoma [58,69]. In addition to being highly expressed in normal salivary gland, *NFIB* also contains several super-enhancers in the 5′- and 3′-parts of the gene and its flanking sequences [37,70], suggesting that enhancer-hijacking events may also contribute to the activation of *PLAG1* and *HMGA2* in PAs [37].

In addition to *NFIB* and *FHIT*, there are at least five additional known fusion partner genes to *HMGA2* in PA, including *FTO*, *HELB*, *TMTC2*, *RPSAP52*, and *WIF1* [71,72,73,74] (Figure 4B and Supplementary Table S1). Available data indicate that the latter, together with *NFIB*, are the two most common fusion partners [37,42,72,74]. *WIF1* is located only 0.7 Mb centromeric to *HMGA2* and the *HMGA2::WIF1* fusion is thus generated by a cryptic intrachromosomal rearrangement [42]. Previous studies have shown that the *HMGA2::WIF1* fusion is also amplified together with several other closely linked genes in 12q. Interestingly, Agaimy and co-workers recently suggested that PAs with *HMGA2::WIF1* fusions are characterized by a prominent trabecular and canalicular adenoma-like morphology [72]. The molecular mechanism by which the fusions activate *HMGA2* is only partly known. In addition to enhancer-hijacking, it has been suggested that binding sites for negatively regulating microRNAs in the 3′-UTR of *HMGA2* are lost in the fusions that may contribute to activation of the gene [18,75]. However, additional studies are needed to fully understand the mechanisms by which *HMGA2* is activated in PA.

## 4. The 8q12 and 12q13-15 Rearrangements in PA Activate the HMGA2-PLAG1-IGF2 Pathway

As discussed in this review, the genomic hallmarks of PA are translocations/rearrangements, with consistent breakpoints in 8q12 and 12q13-15, leading to gene fusions with the oncogenic drivers *PLAG1* and *HMGA2*. However, the molecular association between these two seemingly different aberrations have been unclear. There is now convincing evidence to suggest that *HMGA2* is an upstream regulator of *PLAG1* expression and that HMGA2 regulates the expression of *IGF2* via PLAG1 in a PLAG1-independent manner [68,76]. This new information emanates mainly from recent studies of the Silver–Russell syndrome (SRS) [76], a condition characterized by pre- and postnatal growth retardation associated with IGF2-deficiency [77,78]. Notably, it was recently shown that a subset of SRSs is caused by mutations in *HMGA2* or in the HMGA2 regulated gene *PLAG1* [76,79,80]. Inactivation of either of these genes leads to downregulation of IGF2 and a growth retardation phenotype. We now propose that activation of *HMGA2* or *PLAG1* expression due to chromosome 12q13-15 and 8q12 rearrangements in PA result in upregulation of IGF2 expression and tumorigenesis. This conclusion is also supported by previous studies showing co-expression of *HMGA2* and *PLAG1* in PAs with 12q13-15 rearrangements [19,68]. Taken together, these studies for the first time provide a common explanation for the 8q12 and 12q13-15 aberrations in PA and identifies IGF2 as a major oncogenic driver in PA.

## 5. Clinical Significance of Genomic Alterations in PA

Recurrent PAs may pose a significant clinical problem [81,82,83]. With current management of parotid PAs, the recurrence rate is around 3% [3,83]. Little, if anything, is known about the genomic profile of recurrent PAs. Preliminary cytogenetic analysis of 19 recurrent PAs have shown a similar distribution of 8q12, 12q13-15, and non-recurrent clonal aberrations as in primary non-recurrent tumors after more than 20 years follow-up (unpublished data). Similar observations were made also from an unpublished smaller series of cytogenetically analyzed primary PAs that recurred after 6–36 years. Taken together, these preliminary studies demonstrate that recurrent PAs are genetically stable even after long growth periods in vivo and that they do not deviate from the general pattern of chromosome aberrations in PA.

Malignant transformation of PA may occur in up to 10% of the cases and is increased in recurrent tumors and in tumors with long growth periods in vivo [1]. Studies of the cytogenetic and gene fusion landscape of CXPA is very limited. CXPAs show the PA specific *PLAG1*- or *HMGA2*-fusions as well as a number of other both recurrent and non-recurrent aberrations [42,43,60,84,85]. Previous studies have suggested that PAs with amplification of genes in 12q13-21, including *MDM2*, *HMGA2* or an *HMGA2*-fusion, have an increased risk of malignant transformation [42,43,44]. In addition, amplification and overexpression of the *FGFR1::PLAG1* fusion has been suggested as a potential biomarker for malignant transformation of PA [49].

PAs are characterized by histopathological diversity and there are several other benign and malignant salivary gland tumors that can mimic PA (reviewed in [8]). There is thus a need for diagnostically useful molecular markers to help distinguish PA from its mimics. Importantly, previous studies have shown that aberrations of 8q12 and 12q13-15 involving *PLAG1* and *HMGA2* are specific to PA and CXPA (cf. above) among salivary gland tumors [18,27,84,86,87,88,89]. Aberrations of these genes may be detected by FISH, RT-PCR, immunohistochemistry (IHC), and other molecular techniques such as for example RT-PCR and next generation sequencing. For routine histopathological purposes, FISH and IHC are perhaps most useful and there are several studies showing that PLAG1 IHC is a sensitive marker for PA and CXPA (cf. above).

Today the mainstay treatment of PA is surgery. However, surgery in the parotid and other regions of the head and neck is not uncomplicated with risk of damage to the facial nerve and a number of other complications. Therefore, especially in the recurrent setting with substantial risk for the VIIth nerve, non-surgical treatments are needed, and the recent discovery that the 8q12 and 12q13-15 aberrations lead to activation of the HMGA2-PLAG1-IGF2 pathway opens up new avenues for future medical treatment of PAs with for example IGF2-inhibitors.

## 6. Conclusions

The genomic hallmark of PA is translocations with consistent breakpoints in 8q12 and 12q13-15 resulting in gene fusions involving the transcription factor genes *PLAG1* and *HMGA2*. *PLAG1* is activated by promoter swapping/enhancer hijacking whereas *HMGA2* is activated by gene truncation/enhancer hijacking. Importantly, recent studies have shown that HMGA2 is an upstream regulator of *PLAG1* expression and that HMGA2 regulates the expression of IGF2 via *PLAG1*. This provides a novel explanation for the 8q12 and 12q13-15 aberrations in PA and identifies IGF2 as a major oncogenic driver and therapeutic target in PA. Taken together, these studies have important diagnostic and possible future therapeutic implications for patients with PA.

## Data Availability

Not applicable.

## Supplementary Materials

The following supporting information can be downloaded at: https://www.mdpi.com/article/10.3390/biomedicines10081970/s1. Table S1: Overview of all *PLAG1*- and *HMGA2*-fusions so far described in pleomorphic salivary gland adenomas in the literature. References [11,12,13,17,19,37,38,39,42,60,61,62,71,72,73,74,85,89,90,91] are cited in the supplementary materials.
