# Supplementary material for: Chromosome Translocations, Gene Fusions, and Their Molecular Consequences in Pleomorphic Salivary Gland Adenomas
Source: Biomedicines. 2022 Aug 14;10(8):1970. doi: 10.3390/biomedicines10081970 (PMC9405559; doi:10.3390/biomedicines10081970)
Supplement: Supplementary file 1 [file biomedicines-10-01970-s001.zip › biomedicines-1852371-supplementary.pdf]

Supplementary Table S1. Overview of all *PLAG1*- and *HMGA2*-fusions so far described in pleomorphic salivary gland adenomas in the literature. The fusions were identified by RNA-sequencing, RT-PCR, fluorescence in situ hybridization (FISH), and/or array-based comparative genomic hybridization (arrayCGH).

Gene fusions	No. of cases	References
***PLAG1*-fusions**		
CTNNB1::PLAG1	49	[1-9]
CHCHD7::PLAG1	19	[3,5,7,10]
FGFR1::PLAG1	10	[11]
LIFR::PLAG1	8	[3,5,12]
TCEA1::PLAG1	3	[2,10]
FBXO32::PLAG1	2	[7,13]
NCALD::PLAG1	3	[7]
NTF3::PLAG1	2	[7,13]
ACTA2::PLAG1	1	[7]
DSTN::PLAG1	1	[7]
C1orf116::PLAG1	1	[13]
GEM::PLAG1	1	[13]
BOC::PLAG1	1	[14]
NFIB::PLAG1	1	[6]
***HMGA2*-fusions**		
HMGA2::WIF1	26	[3,5-9,15-17]
HMGA2::NFIB	5	[6,18]
HMGA2::FHIT	1	[19]
HMGA2::RPSAP52	1	[17]
HMGA2::HELB	1	[17]
HMGA2::TMTC2	1	[20]
HMGA2::FTO	1	[8]

References

1. Kas K, Voz ML, Röijer E, Aström AK, Meyen E, Stenman G, et al. Promoter swapping between the genes for a novel zinc finger protein and beta-catenin in pleiomorphic adenomas with t(3;8)(p21;q12) translocations. Nature genetics 1997; 15 (2):170-174.
2. Astrom AK, Voz ML, Kas K, Roijer E, Wedell B, Mandahl N, et al. Conserved mechanism of PLAG1 activation in salivary gland tumors with and without chromosome 8q12 abnormalities: identification of SII as a new fusion partner gene. Cancer research 1999; 59 (4):918-923.

3. Matsuyama A, Hisaoka M, Nagao Y, Hashimoto H. Aberrant PLAG1 expression in pleomorphic adenomas of the salivary gland: a molecular genetic and immunohistochemical study. Virchows Archiv : an international journal of pathology 2011; 458 (5):583-592.
4. Katabi N, Ghossein R, Ho A, Dogan S, Zhang L, Sung YS, et al. Consistent PLAG1 and HMGA2 abnormalities distinguish carcinoma ex-pleomorphic adenoma from its de novo counterparts. Human pathology 2015; 46 (1):26-33.
5. Asahina M, Saito T, Hayashi T, Fukumura Y, Mitani K, Yao T. Clinicopathological effect of PLAG1 fusion genes in pleomorphic adenoma and carcinoma ex pleomorphic adenoma with special emphasis on histological features. Histopathology 2019; 74 (3):514-525.
6. Afshari MK, Fehr A, Nevado PT, Andersson MK, Stenman G. Activation of PLAG1 and HMGA2 by gene fusions involving the transcriptional regulator gene NFIB. Genes, chromosomes & cancer 2020; 59 (11):652-660.
7. Bubola J, MacMillan CM, Demicco EG, Chami RA, Chung CT, Leong I, et al. Targeted RNA sequencing in the routine clinical detection of fusion genes in salivary gland tumors. Genes, chromosomes & cancer 2021; 60 (10):695-708.
8. Sun L, Petrone JS, McNulty SN, Evenson MJ, Zhu X, Robinson JA, et al. Comparison of gene fusion detection methods in salivary gland tumors. Human pathology 2022; 123:1-10.
9. Pareja F, Da Cruz Paula A, Gularte-Mérida R, Vahdatinia M, Li A, Geyer FC, et al. Pleomorphic adenomas and mucoepidermoid carcinomas of the breast are underpinned by fusion genes. NPJ breast cancer 2020; 6:20.
10. Asp J, Persson F, Kost-Alimova M, Stenman G. CHCHD7-PLAG1 and TCEA1-PLAG1 gene fusions resulting from cryptic, intrachromosomal 8q rearrangements in pleomorphic salivary gland adenomas. Genes, chromosomes & cancer 2006; 45 (9):820-828.
11. Persson F, Winnes M, Andrén Y, Wedell B, Dahlenfors R, Asp J, et al. High-resolution array CGH analysis of salivary gland tumors reveals fusion and amplification of the FGFR1 and PLAG1 genes in ring chromosomes. Oncogene 2008; 27 (21):3072-3080.
12. Voz ML, Aström AK, Kas K, Mark J, Stenman G, Van de Ven WJ. The recurrent translocation t(5;8)(p13;q12) in pleomorphic adenomas results in upregulation of PLAG1 gene expression under control of the LIFR promoter. Oncogene 1998; 16 (11):1409-1416.
13. Baneckova M, Uro-Coste E, Ptakova N, Steiner P, Stanowska O, Benincasa G, et al. What is hiding behind S100 protein and SOX10 positive oncocytomas? Oncocytic pleomorphic adenoma and myoepithelioma with novel gene fusions in a subset of cases. Human pathology 2020; 103:52-62.
14. Pei J, Liu JC, Ehya H, Wei S. BOC-PLAG1, a new fusion gene of pleomorphic adenoma: Identified in a fine-needle aspirate by RNA next-generation sequencing. Diagnostic cytopathology 2021; 49 (6):790-792.
15. Queimado L, Lopes CS, Reis AM. WIF1, an inhibitor of the Wnt pathway, is rearranged in salivary gland tumors. Genes, chromosomes & cancer 2007; 46 (3):215-225.
16. Persson F, Andrén Y, Winnes M, Wedell B, Nordkvist A, Gudnadottir G, et al. High-resolution genomic profiling of adenomas and carcinomas of the salivary glands reveals amplification, rearrangement, and fusion of HMGA2. Genes, chromosomes & cancer 2009; 48 (1):69-82.
17. Agaimy A, Ihrler S, Baněčková M, Costés Martineau V, Mantsopoulos K, Hartmann A, et al. HMGA2-WIF1 Rearrangements Characterize a Distinctive Subset of Salivary Pleomorphic Adenomas With Prominent Trabecular (Canalicular Adenoma-like) Morphology. The American journal of surgical pathology 2022; 46 (2):190-199.
18. Geurts JM, Schoenmakers EF, Röijer E, Aström AK, Stenman G, van de Ven WJ. Identification of NFIB as recurrent translocation partner gene of HMGIC in pleomorphic adenomas. Oncogene 1998; 16 (7):865-872.
19. Geurts JM, Schoenmakers EF, Röijer E, Stenman G, Van de Ven WJ. Expression of reciprocal hybrid transcripts of HMGIC and FHIT in a pleomorphic adenoma of the parotid gland. Cancer research 1997; 57 (1):13-17.
20. Wasserman JK, Dickson BC, Smith A, Swanson D, Purgina BM, Weinreb I. Metastasizing Pleomorphic Adenoma: Recurrent PLAG1/HMGA2 Rearrangements and Identification of a Novel HMGA2-TMTC2 Fusion. The American journal of surgical pathology 2019; 43 (8):1145-1151.
